# Smartphone language and resting‐state EEG indicators of self‐focused attention prospectively predict major depressive disorder risk in adolescents

**DOI:** 10.1111/jcpp.70096

**Published:** 2025-12-15

**Authors:** Lilian Y. Li, Nayoung Kim, Esha Trivedi, Sarah E. Sarkas, Madeline M. McGregor, Aishwarya Sritharan, Katherine Durham, Ivan Alekseichuk, Allison M. Letkiewicz, Vijay A. Mittal, David Pagliaccio, Nicholas B. Allen, Randy P. Auerbach, Stewart A. Shankman

**Affiliations:** ^1^ Department of Psychiatry and Behavioral Sciences, Stephen M. Stahl Center for Psychiatric Neuroscience Northwestern University Chicago IL USA; ^2^ Department of Psychiatry Columbia University New York NY USA; ^3^ Division of Child and Adolescent Psychiatry New York State Psychiatric Institute New York NY USA; ^4^ Department of Psychology University of Oregon Eugene OR USA

**Keywords:** Depression, adolescence, self‐focused attention, digital phenotyping, EEG alpha oscillations

## Abstract

**Background:**

Central to major depressive disorder (MDD) onset and maintenance is maladaptive self‐focused attention, which can be reliably indexed by greater: (a) usage of first‐person singular pronouns (e.g., *I*) in natural language and (b) alpha oscillations in resting‐state EEG. Integrating these largely parallel bodies of research, the present study sought to explicate the associations between, and prospective predictive utility of, linguistic and neural indicators of self‐focused attention in adolescents with remitted MDD over 12 months.

**Methods:**

At baseline, 126 adolescents (ages 13–18) with (*n* = 66) and without (*n* = 60) remitted MDD completed resting‐state EEG. Retrospective interviews determined the occurrence of major depressive episodes (MDEs) during the follow‐up period. A total of ~2.3 million messages were passively acquired from adolescents' smartphones, on which the proportion of first‐person singular pronouns was derived.

**Results:**

During the 12 months, 29 (23.0%) participants developed an MDE (28 remitted MDD, 1 control). Cox regression showed that while greater usage of first‐person singular pronouns prior to MDE increased the risk for MDE (hazard ratio [HR] = 2.02, *p* < .001), greater resting‐state alpha power at baseline decreased the risk for MDE (HR = 0.78, *p* = .001). Moreover, greater alpha power predicted subsequent first‐person singular pronoun usage (*β* = 0.17, *p* = .004). Mediation analysis indicated a marginal suppression effect (bootstrapped indirect effect *p* < .10), such that accounting for first‐person singular pronoun usage amplified the association between alpha power and MDE risk.

**Conclusions:**

Findings highlight functionally distinct alpha mechanisms and provide support for smartphone‐based first‐person singular pronoun usage as a neurobehavioral risk factor and a potentially promising intervention target for adolescent MDD.

The high and rising rates of major depressive disorder (MDD) among adolescents (SAMHSA, 2023) and the often‐recurrent course that characterizes MDD (Buckman et al., 2018) create a substantial personal and societal burden worldwide (Ward & Goldie, 2024). Thus, there is a major public health need to identify risk markers of MDD that persist into remission. A core feature of depression is a pervasive, and often automatic, negative bias, much of which is focused on the self (Beck & Haigh, 2014; Pyszczynski et al., 1987). Indeed, excessive self‐focused attention is a relatively stable (i.e., state‐independent) risk factor linked to depression onset and maintenance (Mor & Winquist, 2002; Phillips, Hine, & Thorsteinsson, 2010). Consequently, clarifying the neurobehavioral mechanisms of self‐focused attention that confer risk for adolescent depression could aid in the development of novel targets for the prevention of MDD onset and recurrence.

Prevailing theories indicate that the association between self‐focused attention and depression stems from a maladaptive self‐regulatory cycle (Carver & Scheier, 1998; Duval & Wicklund, 1972; Ingram, 1990; Pyszczynski et al., 1987). Specifically, attention to the self triggers an evaluative process whereby the current self is compared to a salient standard. If the current self falls short of the standard, individuals will engage in a cycle of discrepancy‐reducing behaviors and evaluations that last until a match is obtained. Negative affect, and depression in particular, is thought to result from one trying to reduce an irreducible discrepancy and thus being stuck in this self‐regulatory cycle (Carver & Scheier, 1998; Duval & Wicklund, 1972; Ingram, 1990; Pyszczynski et al., 1987). That is to say, self‐focused attention, in and of itself, is not maladaptive; it is the perseveration on self‐relevant information, particularly of negative nature, that contributes to depression and other psychopathologies (Ingram, 1990; Pyszczynski et al., 1987). In support, meta‐analytic findings demonstrate that elevated self‐focused attention, predominantly measured via self‐reports, is associated with elevated negative affect and depression, with this association being stronger following negative (vs. positive) events and focusing on negative (vs. positive) aspects of the self (Mor & Winquist, 2002).

In addition to self‐reports, self‐focused attention can also be reliably indexed by the use of certain linguistic features; that is, first‐person singular pronouns such as *I*, *me*, and *mine* (Ireland & Mehl, 2014). For example, early work using experimental manipulations showed that induced self‐focus (e.g., placing a mirror in front of participants) resulted in greater first‐person singular pronoun usage (Davis & Brock, 1975). Moreover, having participants write an essay using first‐person singular pronouns (vs. third‐person) resulted in greater self‐focus (Fenigstein & Levine, 1984; for a review, see: Mor & Winquist, 2002). Importantly, greater usage of first‐person singular pronouns in a variety of natural language data (e.g., personal essays, interviews) is cross‐sectionally associated with greater depression severity (Edwards & Holtzman, 2017; Tackman et al., 2019), with recent evidence particularly implicating first‐person singular pronouns in negatively valenced text (Collins et al., 2025). On the other hand, explicitly reducing first‐person singular pronoun usage when reflecting on one's emotions dampens negative emotion reactivity (Kross et al., 2014; Moser et al., 2017).

Accumulating research has also expanded the study of first‐person singular pronouns into the digital space. In line with laboratory work, studies demonstrate that naturalistic usage of first‐person singular pronouns extracted from social media posts (Eichstaedt et al., 2018; Kelley & Gillan, 2022) and, more recently, from smartphones (Funkhouser et al., 2024; Li et al., 2023; McNeilly et al., 2023, 2024) relates to depression. Recent work from our team further observed that greater usage of first‐person singular pronouns in smartphone social communication prospectively predicted a greater likelihood of major depressive episode up to 6 weeks in advance (Funkhouser et al., 2024). Given the ubiquity of smartphone use among youth (Rideout, Peebles, Mann, & Robb, 2022), tracking first‐person singular pronoun usage on the smartphone could offer a prime window into understanding and intervening on self‐focused attention in adolescent depression at large scale. Nevertheless, a critical issue to consider is that language data are inherently noisy, which has likely contributed to the small effect sizes observed in association with depression (Tackman et al., 2019). This limitation also reduces the confidence in drawing the conclusion that it is self‐focused attention, rather than noise or some other processes that result in the observed association. Shedding light on the neural correlates of smartphone‐based first‐person singular pronoun usage using high‐quality laboratory data is therefore a key step to bolster the validity of this linguistic marker.

Although the neurobiological mechanisms underlying first‐person singular pronoun usage remain poorly understood, abundant research has delineated the neural correlates of self‐focused attention broadly. This research using EEG has particularly implicated the alpha oscillations (8–13 Hz), which are maximal over posterior regions during quiet wakefulness (Klimesch, Sauseng, & Hanslmayr, 2007; Knyazev, 2013; Sadaghiani & Kleinschmidt, 2016). Alpha oscillations have been suggested to be the primary rhythm for intrinsic (i.e., self‐focused) attention via inhibiting bottom‐up sensory processing (Klimesch et al., 2007; Sadaghiani & Kleinschmidt, 2016). Specifically, greater resting‐state alpha activity is correlated with greater degrees of self‐referential thoughts as well as greater activity within the default mode network (DMN), a key mediator of self‐referential processing, in simultaneous EEG‐fMRI recordings (for a review, see: Knyazev, 2013). Beyond correlational evidence, transcranial alpha stimulation at occipitoparietal regions has been shown to enhance DMN connectivity (Clancy et al., 2022). In short, increased resting‐state alpha activity is a well‐validated neural indicator for increased self‐focused attention.

Interestingly, despite robust links with self‐focused attention, the association between resting‐state alpha activity and depression has been mixed, with studies showing enhanced (Grin‐Yatsenko, Baas, Ponomarev, & Kropotov, 2010; Pollock & Schneider, 1990), reduced (Jiang et al., 2016; Umemoto et al., 2021; Volf & Passynkova, 2002; Zoon et al., 2013), and equivocal (Ray et al., 2023) levels of alpha power in those with MDD compared to controls. This discrepancy likely stems from the fact that alpha oscillations are generated by several distinct mechanisms, supporting a broad range of adaptive and maladaptive functions (Klimesch et al., 2007; Knyazev, 2013; Sadaghiani & Kleinschmidt, 2016). In particular, alpha oscillations have also been implicated in cognitive control functions (Klimesch et al., 2007; Sadaghiani & Kleinschmidt, 2016) that may serve a protective role against depression. Accordingly, more work, especially those employing longitudinal models, is needed to disentangle the distinct roles of resting‐state alpha power in depression.

Using a longitudinal sample of adolescents with remitted MDD and healthy controls, the present study tested whether linguistic and neural indicators of self‐focused attention predicted MDD onset and recurrence. Participants completed resting‐state EEG at baseline and were followed for 12 months, during which all keyboard inputs into their own smartphones were passively collected. Our study had four objectives. First, we sought to replicate prior findings on the prospective association between first‐person singular pronoun usage and MDD risk. Second, we explored whether resting‐state alpha power predicted MDD risk. Third, to examine convergent validity, we tested the association between first‐person singular pronoun usage and resting‐state alpha power. Last, to possibly explain prior mixed findings on alpha power and depression, we explored whether accounting for maladaptive self‐focused attention as indexed by first‐person singular pronoun usage could augment the protective role of alpha power. Findings generated from this study hold promise for clarifying the neural correlates of linguistic self‐focused attention and MDD risk, a key step toward the development of neuroscience‐informed tools that would advance the clinical translation of basic findings on self‐focused attention.

## Methods

### Participants

Adolescents aged 13–18 years old were recruited from the community and mental health clinics in New York, NY, and Chicago, IL, areas from September 2020 to October 2023 as part of a larger, ongoing longitudinal project (for recruitment details, see: Funkhouser et al., 2024; Li et al., 2023). General inclusion criteria relevant to this study included: (a) fluency in English, (b) Wechsler Abbreviated Scale of Intelligence‐II (WASI‐II; Wechsler, 2011) > 85, (c) ownership of a personal smartphone (Android or iOS), and (d) right‐handedness. General exclusion criteria included: (a) current moderate or severe substance use disorder and (b) lifetime history of bipolar or psychotic disorders, oppositional defiant disorder, conduct disorder, organic mental disorder, or developmental disorder.

The 163 participants who completed at least one follow‐up (i.e., 6‐month or 12‐month) were considered for inclusion in the current study. Of the 163 participants, 37 (22.7%) were excluded due to: (a) missing resting‐state EEG data (*n* = 8), (b) poor EEG data quality (*n* = 11), (c) missing keyboard input data due to technical difficulties and/or withdrawal from the mobile sensing portion of the study (*n* = 6), and (d) having fewer than 500 words in their keyboard input data (*n* = 12). The final sample (*N* = 126) included 66 adolescents with lifetime, but not current, MDD (i.e., remitted MDD) and 60 adolescents without a lifetime history of psychiatric disorders (i.e., healthy control).^1^ For the remitted MDD group, the average age of onset was 12.68 years (*SD* = 2.88, range = 6–17), the average number of lifetime episodes was 3.00 (*SD* = 2.67, range = 1–15), and the average number of days since the last episode was 496.11 days (*SD* = 520.51, range = 21–2,453). Included and excluded participants did not differ in any demographic and clinical characteristics, except that the excluded (vs. included) group had more: (a) non‐White participants and (b) participants from New York City (see Table S1).

### Procedure and materials

At baseline, trained interviewers administered: (a) the DSM‐5 version of the Kiddie Schedule for Affective Disorders and Schizophrenia (K‐SADS‐PL; Kaufman et al., 1997) to assess the lifetime presence of psychiatric disorders (*κ* = .98), (b) the WASI‐II (two‐subtest form) to assess verbal intelligence (Vocabulary subtest) and nonverbal intelligence (Matrix Reasoning subtest), and (c) the Children's Depression Rating Scale–Revised (CDRS‐R; Poznanski & Mokros, 1996) to assess depression symptom severity. In a separate baseline session, participants completed resting‐state EEG. Next, participants installed the Effortless Assessment Research System (Lind et al., 2023) app onto their personal smartphones to collect naturalistic keyboard inputs over the 12‐month follow‐up period. They were instructed to keep the app running while using their phone as they normally would.

Approximately 6 and 12 months after the baseline interview, participants completed the Adolescent Longitudinal Interval Follow‐Up Evaluation (A‐LIFE; Keller et al., 1987), a timeline follow‐back interview assessing weekly MDD severity during the prior 6 months. Almost all participants (*n* = 125; 99.2%) completed the 6‐month A‐LIFE,^2^ and 101 (80.2%) completed the 12‐month (*n* = 11 had not yet reached their 12‐month follow‐up; *n* = 14 lost to follow‐up). The average follow‐up time is 48.50 weeks (*SD* = 10.77, range = 26–71).

All study procedures were carried out in accordance with the Declaration of Helsinki and approved by the New York State Psychiatric Institute Institutional Review Board. Informed assent and consent were obtained from the adolescent and parent, respectively, and 18‐year‐old adolescents provided consent.

#### Clinical interviews

At baseline, current depression symptom severity was assessed with the CDRS‐R (Poznanski & Mokros, 1996). The CDRS‐R is a 17‐item semistructured interview that assesses symptoms of depression over the past 2 weeks, with greater scores indicating greater severity (*α* = .75). Raw scores were then converted to *T* scores.

At the 6‐ and 12‐month follow‐up assessments, weekly MDD ratings were retrospectively assessed with the A‐LIFE (Keller et al., 1987). Participants were reminded of MDD symptoms and severity reported at their previous assessment and identified change points in MDD severity since the previous assessment. Time anchors (e.g., holidays, birthdays, and school breaks) were used to improve recall. For each week since the previous assessment, interviewers assigned psychiatric status ratings (PSRs) using a scale that ranged from 1 (*no symptoms present*) to 6 (*full‐threshold with extreme impairment/distress*), with PSR ≥4 indicating full‐threshold MDD. Participants with at least two consecutive weeks of PSR ≥4 were coded as having a major depressive episode (MDE) during the follow‐up period (Kaufman et al., 1997), with the cutoff date defined as the first day of their first MDE. For participants not having an MDE during the follow‐up, the cutoff date was defined as the day of their last follow‐up assessment. The number of days between the cutoff date and baseline was then calculated.

#### EEG recording and processing

At baseline, resting‐state EEG (3‐min eyes open and 3‐min eyes closed) was acquired with the 32‐channel ActiCHamp system (Brain Products, Munich, Germany) at a sampling rate of 1,000 Hz. All channels were referenced online to FCz, with impedances kept below 20 kΩ. Offline signal processing was conducted in MATLAB following established pipelines (Miyakoshi, n.d.) (see Appendix S1 for details).

After preprocessing, 4‐s epochs with 50% overlap were extracted from the eyes‐closed condition (when alpha power is maximal; Klimesch et al., 2007; Knyazev, 2013; Sadaghiani & Kleinschmidt, 2016), with baseline correction applied to the entire epoch (overlapping window used to accommodate edge artifact). Then, for each epoch and channel, the artifact‐free EEG signal was band‐pass filtered at 8–13 Hz (alpha band), Hilbertized, and the first and last 1‐s portions removed, yielding 2‐s nonoverlapping epochs. On average, participants had 79.60 epochs (*SD* = 9.81, range = 44–87). Power was calculated as the squared amplitude, log transformed, and averaged across epochs and channels to yield whole‐brain estimates.^3^

#### Keyboard input data

Keyboard inputs across all smartphone apps from the consenting participant's device were passively collected via a keyboard logger and were continuously encrypted and uploaded to a secure cloud computing service for storage (see Appendix S1 for further discussions on protection of privacy and ethical research practices). Each keyboard input was stamped with the date and time, as well as the application being used in the foreground. Our research team then downloaded and unencrypted the keyboard input data, which were subsequently transformed into complete words and messages using a custom script that identifies logical divisions of writing (e.g., long pauses, changes in app).

Basic preprocessing steps were first applied to improve text analysis accuracy, following previous research (Li, Schiffman, & Martin, 2022; Silge & Robinson, 2017). Preprocessing included: (a) removing URLs and mentions (e.g., @JohnSmith), (b) extracting hashtag contents and, if necessary, separating hashtags into words, (c) removing elongated tails (e.g., YAYYYY to YAY), and (d) removing non‐English messages (0.3%).

**Figure 1:**
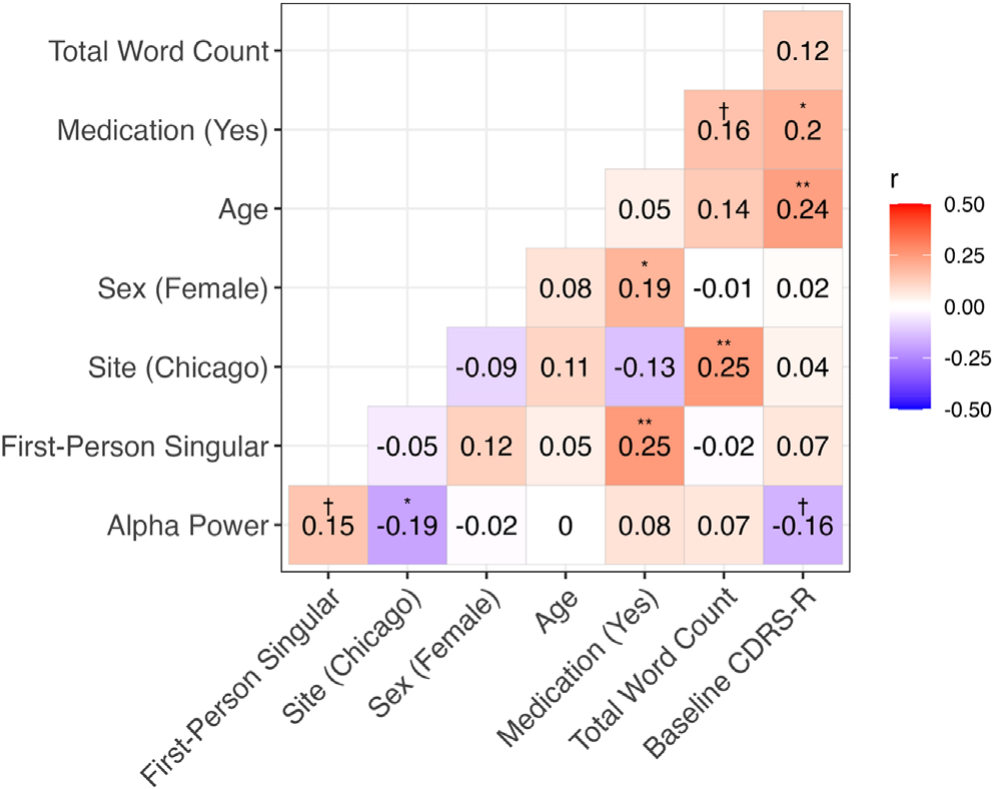
Bivariate correlations between predictors and potential covariates. CDRS‐R, Children's Depression Rating Scale–Revised. ^†^ *p* < .10, * *p* < .05, ** *p* < .01

The Linguistic Inquiry and Word Count (Pennebaker, Booth, Boyd, & Francis, 2015) was used to estimate the proportion of pronouns in each message, including first‐person singular, first‐person plural, second person, third‐person singular, and third‐person plural. Linguistic self‐focused attention score was then calculated as the proportion of first‐person singular pronouns adjusted by the overall pronoun usage in each message [i.e., first‐person singular/(first‐person singular + first‐person plural + second‐person + third‐person singular + third‐person plural)] (Nook, Hull, Nock, & Somerville, 2022). To establish a clear temporal sequence between linguistic self‐focused attention and MDE, only messages prior to the occurrence of the MDE during the follow‐up were included (for those who did not develop an MDE, their full keyboard input data were used). Further, to yield a reliable estimate, participants with fewer than 500 words in their total word count prior to the cutoff date were excluded, following prior recommendations (Eichstaedt et al., 2018; Kern et al., 2016). For each participant, an overall linguistic self‐focused attention score was then calculated by averaging across all messages. Of note, those who did (vs. did not) develop an MDE during the follow‐up did not significantly differ in their volume of keyboard input data (number of messages: *t*(124) = −0.94, *p* = .35; word count: *t*(124) = −1.04, *p* = .30). The volume of keyboard input data was also not related to linguistic self‐focused attention scores (see Figure 1 for bivariate correlations between predictors and potential covariates).

### Statistical analyses

Preliminary analyses examined the association between risk for MDE and various demographic, clinical, and study characteristics for their potential inclusion as covariates. Female sex (hazard ratio [HR] = 3.50, *p* < .001), older age (HR = 1.15, *p* < .001), and taking (vs. not taking) psychiatric medication at baseline (HR = 4.30, *p* = .034) increased the risk for MDE during follow‐up. However, when sex, age, and medication status were entered in the same model, only sex remained significant and was thus retained as a covariate in the analyses described below. In addition, all analyses included the covariate of baseline depression symptom severity (CDRS‐R) and robust standard errors correcting for clustering of participants within sites.

To examine the effect of linguistic and neural indicators of self‐focused attention on the risk for MDE during follow‐up, a Cox proportional hazards model was separately conducted for first‐person singular pronouns and alpha power predicting time to MDE. To examine the association between linguistic and neural indicators of self‐focused attention, a linear regression model was conducted, with first‐person singular pronouns as the outcome and alpha power as the predictor. Last, we examined a mediation model of alpha power, first‐person singular pronouns, and the risk for MDE. The direct effect of alpha power on the risk for MDE was tested using a Cox proportional hazards model, controlling for first‐person singular pronoun usage. The indirect effect was tested using bootstrapped 95% confidence intervals (*n* = 10,000). All analyses were performed in R, version 4.4.3 (R Core Team, 2025).

## Results

### Sample characteristics

Table 1 summarizes the participant demographic and clinical information. Participants were primarily female and most identified as cisgender, but with diverse racial, ethnic, and socioeconomic backgrounds. Among the 126 participants, 29 (23.0%) developed an MDE during the 12 months, consisting of 28 (42.4%) from the remitted MDD group and 1 (1.7%) from the healthy control group. The average time to first MDE was 146.76 days (*SD* = 97.62, range = 15–372). During the period preceding their cutoff date (MDE or last follow‐up date), a total of 2,355,587 messages and 12,543,068 words were collected, averaging 18,695.13 messages and 99,548.16 words per person.

**Table 1:** Demographic and clinical characteristics

Characteristic	*N* = 126
Demographic	
Sex (female) *n* (%)	93 (73.8)
Cisgender *n* (%)	117 (92.8)
Age *M* (*SD*)	16.30 (1.49)
Race/Ethnicity *n* (%)	
White	51 (40.5)
Hispanic	38 (30.2)
Asian	22 (17.5)
Black	9 (7.1)
Biracial/Multiracial	6 (4.8)
Annual household income *n* (%)^a^	
<$24,999	6 (5.7)
$25,000–$49,999	13 (12.3)
$50,000–$74,999	8 (7.5)
$75,000–$99,999	18 (17.0)
≥$100,000	61 (57.5)
Site (Chicago) *n* (%)	71 (56.3)
Follow‐up length (weeks) *M* (*SD*)	48.50 (10.77)
Clinical	
Diagnosis *n* (%)	
Remitted MDD	66 (52.4)
No lifetime diagnosis	60 (47.6)
Had an MDE during follow‐up *n* (%)	29 (23.0)
Taking psychiatric medication at baseline *n* (%)	33 (26.2)
Baseline CDRS‐R *T* score *M* (*SD*)	38.45 (8.69)
Baseline WASI‐II Vocabulary *T* score *M* (*SD*)	62.13 (8.62)
Keyboard input	
Number of messages *M* (*SD*)	18,695.13 (27,627.09)
Total word count *M* (*SD*)	99,548.16 (170,774.95)
Proportion of first‐person singular pronouns *M* (*SD*)	0.56 (0.066)

CDRS‐R, Children's Depression Rating Scale–Revised; MDD, major depressive disorder; MDE, major depressive episode; WASI‐II, Wechsler Abbreviated Scale of Intelligence‐II.

^a^ Household income missing/unknown for 20 participants.

### Prospective predictors of risk for MDE during follow‐up

Greater usage of first‐person singular pronouns prior to MDE significantly increased the risk for MDE during follow‐up, HR = 2.02, 95% CI = [1.39, 2.95], *p* < .001 (see Table S2 for full model outputs and Figure S1 for survival curves).

**Figure 2:**
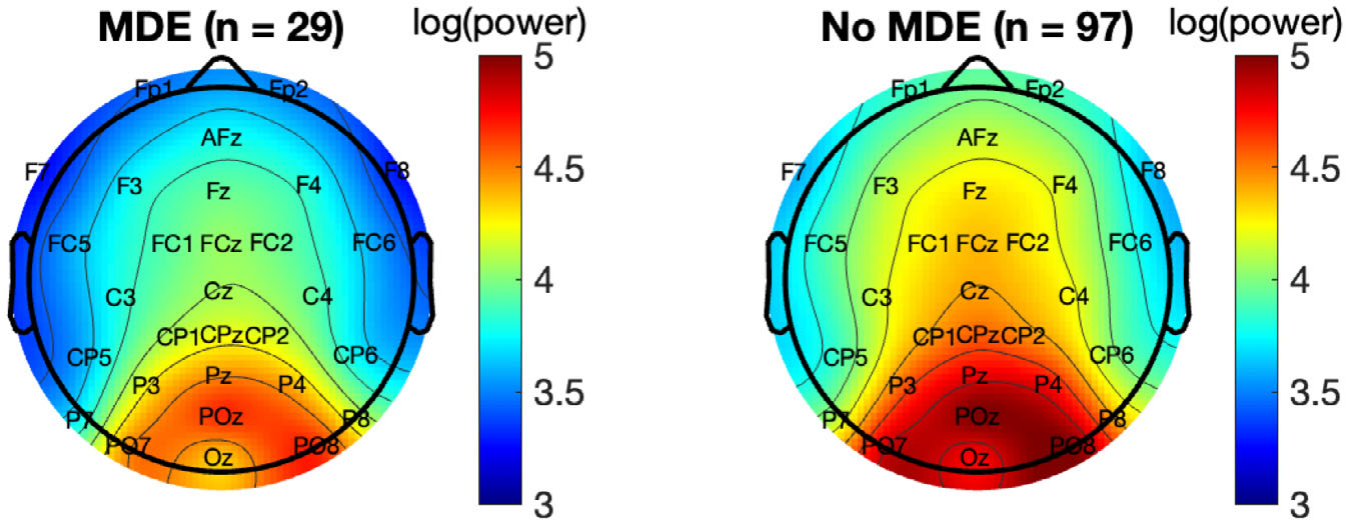
Topographic map of log transformed alpha power during eyes‐closed resting state at baseline by major depressive episode status during follow‐up. MDE, major depressive episode

Greater baseline alpha power significantly decreased the risk for MDE during follow‐up, HR = 0.78, 95% CI = [0.68, 0.91], *p* = .001 (see Figure 2 for topographical maps; for full model outputs and survival curves, see Table S2 and Figure S1). In other words, lower alpha power increased the risk for MDE.

### Alpha power predicted first‐person singular pronoun usage

Greater alpha power significantly predicted greater first‐person singular pronoun usage, *β* = 0.17, 95% CI = [0.055, 0.29], *p* = .004 (see Table S3 for full model outputs).

### Mediation model

**Figure 3:**
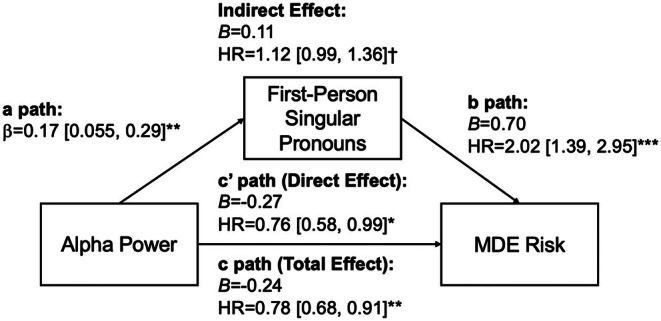
Mediation model for the association between resting‐state alpha power and risk for major depressive episode, mediated by first‐person singular pronoun usage. All models included the covariates of sex and baseline CDRS‐R depression score and cluster robust standard errors. HR, hazard ratio. ^†^ *p* < .10, *p < .05, **p < .01, ***p < .001

A mediation model was conducted with alpha power at baseline as the predictor, first‐person singular pronoun usage from baseline to the day prior to the first MDE as the mediator, and risk for MDE during the 12‐month follow‐up as the outcome (see Figure 3). The direct effect of alpha power (i.e., controlling for first‐person singular pronoun usage; c' path) on risk for MDE remained significant, HR = 0.76, 95% CI = [0.58, 0.99], *p* = .041, which was stronger than the total effect for which first‐person singular pronoun usage was not accounted (c path). The indirect effect of alpha power on risk for MDE approached significance, HR = 1.12, 95% CI = [0.99, 1.36], *p* < .10. These findings suggest a weak suppression effect from first‐person singular pronoun usage in the association between alpha power and risk for MDE.

To further clarify whether self‐focused attention on negatively (vs. positively) valenced information is particularly related to depression, we explored the role of first‐person singular pronoun usage calculated only in negatively vs. positively valenced messages. Overall, mediation models yielded similar results as the main analyses. Although the indirect effect was not statistically significant for either negative or positive messages, the effect sizes (a, b, and c' path) were stronger for negative than positive messages (see Figure S2).

## Discussion

The present study investigated the associations between, and prospective predictive utility of, linguistic and neural indicators of self‐focused attention in adolescent remitted MDD over a 12‐month period. Findings showed that resting‐state alpha power predicted subsequent first‐person singular pronoun usage passively captured from the smartphone, providing convergent validity for smartphone‐based linguistic indicators of self‐focused attention. Interestingly, while both first‐person singular pronoun usage and alpha power prospectively predicted MDD onset and recurrence, they did so in opposite directions, predicting greater and lower risk for MDE, respectively. The mediation model further revealed a suppression effect, albeit weak and approaching significance, such that accounting for first‐person singular pronoun usage enhanced the protective effect of alpha power. Overall, this study provided novel evidence supporting smartphone‐based first‐person singular pronoun usage as a neurobehavioral risk factor for adolescent MDD, demonstrating the potential of passive mobile sensing in translating neuroscientific insights into clinical treatments.

We showed that greater usage of first‐person singular pronouns preceding the MDE predicted greater risk for MDE during a 12‐month follow‐up period. This finding is consistent with the broad literature suggesting that first‐person singular pronoun is a fundamental linguistic marker of depression and other related psychopathologies (Edwards & Holtzman, 2017; Ireland & Mehl, 2014; Tackman et al., 2019). We extended this literature by demonstrating the feasibility of capturing naturalistic first‐person singular pronoun usage on adolescents' smartphones in a long‐term longitudinal design. Of crucial importance, we were the first to explicate the EEG mechanism for this important linguistic marker. That is, greater usage of first‐person singular pronouns was predicted by greater baseline resting‐state alpha power, a frequency band long considered to underpin self‐focused attention (Knyazev, 2013). Moreover, this finding corresponds well with the only other study investigating the neural correlates of smartphone language in a small sample of 40 adolescents with and without MDD (McNeilly et al., 2024). Using resting‐state fMRI, they found that greater DMN and central executive network (CEN) connectivity at baseline predicted greater usage of first‐person singular pronouns collected over an approximately 2‐month period 1 year after the baseline assessment (McNeilly et al., 2024). Collectively, the corresponding EEG and fMRI evidence lends strong support for greater first‐person singular pronoun usage on the smartphone as reflecting excessive self‐focused attention.

On the other hand, the neural indicator of self‐focused attention—resting‐state alpha power—was found to predict *lower* risk for MDE during follow‐up. Explaining this result requires the understanding that scalp‐recorded alpha activity sums over multiple distinct alpha rhythms (Klimesch et al., 2007; Knyazev, 2013; Sadaghiani & Kleinschmidt, 2016). Indeed, simultaneous EEG‐fMRI and source localization of EEG recording during resting state typically observe several brain networks associated with the alpha activity, including the DMN involved in self‐reference as well as the cingulo‐opercular (CO) and fronto‐parietal networks involved in cognitive control functions (Chen, Ros, & Gruzelier, 2013; Knyazev, 2013; Sadaghiani & Kleinschmidt, 2016). With respect to the latter, the alpha rhythm is suggested to exert information gating via inhibitory top‐down control, creating a state of readiness for later task demands (Klimesch et al., 2007; Sadaghiani & Kleinschmidt, 2016). Evidence supporting this view comes from studies showing that greater resting‐state alpha power, particularly over anterior regions, is associated with higher intelligence and better performance on various complex cognitive tasks (Klimesch et al., 2007). Combining distinct functions of alpha oscillations, researchers have hypothesized that the DMN delivers a stream of internal thoughts that is sustained by the control networks that buffer against disruption by external information (Smallwood, Brown, Baird, & Schooler, 2012). Although the current study is unable to tease apart these distinct mechanisms of alpha, work from our team using source localization and effective connectivity on resting‐state alpha based on a subset of the current participants has provided additional insights (Kim et al., 2025). Specifically, we observed hypoconnectivity from midcingulate cortex to precuneus, prominent nodes in the CO and DMN networks, related to greater increases in depressive symptoms from baseline to 6‐month follow‐up (Kim et al., 2025). Thus, our findings suggest that lower resting‐state alpha power may indicate weakened inhibition of irrelevant information, contributing to MDD onset and recurrence.

As such, it is perhaps not surprising that we uncovered a trend for a suppression effect in the mediation model of alpha power, first‐person singular pronoun usage, and risk for MDE. That is, accounting for the effects of maladaptive self‐focused attention somewhat amplified alpha power's role in adaptive top‐down control, which then lowered risk for future MDE. This nonstatistically significant suppression effect was numerically stronger for first‐person singular pronoun usage in negative versus positive messages, providing further support. Although preliminary and warranting future replication, our suggestive evidence of a suppression effect aligns well with another prior study showing a similar suppression effect between baseline depression symptom severity, baseline CEN connectivity (which the authors argued their CEN overlaps with DMN functioning), and smartphone‐based first‐person singular pronoun usage (McNeilly et al., 2024). Extending this prior finding on the cross‐sectional associations, we were able to establish a clear temporal sequence to predict future changes in MDD risk.

There are several limitations of the current study that warrant consideration. First, smartphone language is inherently noisy, which could have contributed to the small effect sizes. In particular, rather than limiting to apps for social communication (e.g., social media, instant messaging) as previously employed (Funkhouser et al., 2024; Li et al., 2023), we included messages across all app platforms. This decision likely further contributed noise to the language data and made it difficult to clarify the context under which first‐person singular pronouns were used.^4^ However, this inclusive approach can also be taken as a strength that demonstrates the generalizability of findings beyond social communication. Relatedly, our use of overall alpha power likely diluted effects that may be specific to narrower regions and frequency ranges. For example, there is evidence that the upper alpha range (10–13 Hz) and in anterior sites are particularly related to cognitive control (Klimesch et al., 2007; Sadaghiani & Kleinschmidt, 2016). Further research employing more refined analyses, such as testing alpha sub‐bands, incorporating alpha power and phase (which are functionally dissociable), and source localization, is needed to further shed light on current findings. Last, although our participants were racially, ethnically, and socioeconomically diverse, we had to disproportionally exclude non‐White (vs. White) participants, primarily due to poor EEG data quality (e.g., braided hair leading to unusable data). While the topic of improving EEG signal quality in non‐White populations is beyond the scope of our study, future research should be mindful of best practices in EEG preparation to foster the inclusivity of neuroscience research (e.g., using dry comb electrodes, including hair type and gel volume as covariates; Adams et al., 2024; Lees, Ram, Swingler, & Gatzke‐Kopp, 2024).

Limitations notwithstanding, current findings have clinical implications for the assessment and treatment of adolescent depression. With respect to assessment, the passive collection of smartphone language data, the inclusive approach to app selection, and the fully automated extraction of first‐person singular pronouns open the window for monitoring adolescent depression in real time and in their natural environments. With respect to treatment, accumulating research indicates that reducing first‐person singular pronoun usage dampens negative emotions (Kross et al., 2014; Moser et al., 2017). One promising strategy to reduce first‐person singular pronoun usage is third‐person self‐talk—using one's own name rather than the first‐person pronoun *I* to refer to the self during introspection (e.g., what is *John* feeling right now vs. what am *I* feeling right now). Third‐person self‐talk has been shown to automatically create the psychological distance needed for adaptive self‐reflection and better emotion regulation (Kross et al., 2014; Moser et al., 2017). This, combined with passive monitoring, could point to a system that identifies windows of excessive self‐focus, for which targeted intervention strategies could be delivered, potentially delaying and preventing a recurrent episode.

## Conclusion

In conclusion, this study combined naturalistic assessment of linguistic self‐focused attention on the smartphone with tightly controlled laboratory assessment of neural self‐focused attention into a longitudinal design. Findings provided novel insights on distinct alpha mechanisms contributing to MDD onset and recurrence in adolescents. Importantly, smartphone‐based first‐person singular pronouns were found to relate to expected neural mechanisms of self‐focused attention and prospectively predict risk for MDE among adolescents with remitted MDD. Consequently, it shows promise as a scalable and actionable risk marker for adolescent MDD and an important first step for clinical translation using smartphones.

## Ethical considerations

This study was approved by the New York State Psychiatric Institute Institutional Review Board on November 6, 2019 (Protocol 7875). Informed assent and consent were obtained from the adolescent and parent, respectively, and 18‐year‐old adolescents provided consent.

**Key points**

**What's known?**

- Excessive self‐focused attention is a key risk factor for depression onset and maintenance and can be reliably indexed by greater: (a) first‐person singular pronoun usage and (b) resting‐state alpha power.

**What's new?**

- Following adolescents with and without remitted MDD for 12 months, greater alpha power at baseline predicted subsequent usage of first‐person singular pronouns passively collected over adolescents' own smartphones.
- Greater first‐person singular pronoun usage and alpha power, respectively, predicted greater and lower risk for major depressive episode (MDE).
- Mediation analysis revealed a marginal suppression effect from first‐person singular pronoun usage in the association between alpha power and MDE risk, indicating distinct alpha mechanisms that are differentially related to depression risk.

**What's relevant?**

- Passive monitoring of first‐person singular pronoun usage could be a promising neuroscience‐informed approach for the identification and intervention of adolescent depression.

## Supporting information


**Appendix S1.** Supplemental method.
**Table S1.** Demographic and clinical characteristics by inclusion status.
**Appendix S2.** Supplemental results.
**Figure S1.** Survival curve for major depressive episode during follow‐up.
**Table S2.** Prospective predictors of risk for major depressive episode.
**Table S3.** Baseline alpha power predicting first‐person singular pronoun usage.
**Figure S2.** Mediation model for the association between resting state alpha power and risk for major depressive episode, mediated by first‐person singular pronoun usage in positive vs. negative messages.

## Data Availability

Deidentified data analytic code used for our primary analyses is available online at the Open Science Framework (https://osf.io/tkrsf/?view_only=592e589ced0a4e1583fc544539ce9643).

## Endnotes

1 Using a subset of the remitted MDD and healthy control participants included in this study (57.9%) and with a follow‐up length of approximately 6 months, we previously showed that greater usage of first‐person singular pronouns concurrently and prospectively predicted a greater likelihood of meeting full‐threshold MDD on a weekly level (Funkhouser et al., 2024).

2 One participant completed the 12‐month, but not 6‐month, assessment, so their A‐LIFE interview assessed the entire 12‐month period since baseline.

3 We report findings on whole‐brain alpha power given that alpha power averaged across frontal (Fp1, Fp2, AFz, F7, F3, Fz, F4, and F8), central (FC5, FC1, FCz, FC2, FC6, C3, Cz, C4, CP5, CP1, CPz, CP2, and CP6), and occipital electrodes (P7, P3, Pz, P4, P8, PO7, POz, PO8, and Oz) was highly correlated, *r*s > .91, *p*s < .001.

4 Although in our analyses, over 80% of all messages came from social communication apps.
