# Supplementary material for: Smartphone language and resting‐state EEG indicators of self‐focused attention prospectively predict major depressive disorder risk in adolescents
Source: J Child Psychol Psychiatry. 2025 Dec 15;67(6):941–50. doi: 10.1111/jcpp.70096 (PMC13170627; doi:10.1111/jcpp.70096)
Supplement: Supplementary file 1 — Appendix S1. Supplemental method. Table S1. Demographic and clinical characteristics by inclusion status. Appendix S2. Supplemental results. Figure S1. Survival curve for major depressive episode during follow‐up. Table S2. Prospective predictors of risk for major depressive episode. Table S3. Baseline alpha power predicting first‐person singular pronoun usage. Figure S2. Mediation model for the association between resting state alpha power and risk for major depressive episode, mediated by first‐person singular pronoun usage in positive vs. negative messages. [file JCPP-67-941-s001.docx]

**Smartphone Language and Resting State EEG Indicators of Self-Focused Attention Prospectively Predict Major Depressive Disorder Risk in Adolescents**

**Supporting Information**

**Appendix S1. Supplemental Method**

**EEG Preprocessing**

Off-line analyses were performed in MATLAB using custom scripts and the EEGlab toolbox (Delorme & Makeig, 2004). First, EEG data were referenced to the average mastoids (online reference FCz added back to the data), downsampled to 500 Hz, and the DC offset was removed from each channel. A band-pass filter was then applied from 1-100 Hz. The 60 Hz line noise was removed using the *cleanLineNoise* function, which used a sliding window to adaptively estimate and subtract the line noise component (Bigdely-Shamlo et al., 2015). Next, using the *clean_rawdata* function, artifactual channels were removed, defined as those: (a) containing more than 5 s of flat signal and/or (b) correlating less than .7 with surrounding channels (Kothe & Makeig, 2013). Artifact subspace reconstruction (ASR) (Mullen et al., 2015) was then applied to correct significant noise bursts, also implemented within *clean_rawdata*. ASR is a principal-component-analysis-based (PCA-based) technique in which data within a 500 ms sliding window (window step = 250 ms) were PCA-decomposed. Noisy components, defined as those with variance greater than 20 *SD* above that of the clean portions of the data, were corrected. Further, time windows were removed if more than 25% of the channels contained high-power artifacts, defined as greater than 7 *SD* above the clean power estimates in the channel. All artifactual channels were replaced by whole head spline interpolation. Lastly, independent component analysis (ICA) was implemented to retain brain-related components only, defined as having greater probability to be brain than artifacts according to an automatic IC classifier (*ICLabel*) (Pion-Tonachini et al., 2019).

**Protection of Privacy and Ethical Research Practices**

As key input data collected via the Effortless Assessment Research System (EARS) are highly granular and could reveal potentially sensitive and identifiable information of the participant, we have implemented a number of procedures to address important ethical issues, including informed assent/consent (i.e., transparency and voluntariness) and data protection (i.e., de-identification, encryption, and limited access to potentially identifiable data) (Jacobson et al., 2020).

First, we believe that transparency in the intent and practice behind the EARS app at the point of obtaining informed assent/consent is a critical element of ethical research practices, especially considering that many participants may not be familiar with the concept of mobile sensing (Mohr et al., 2020; Vitak et al., 2016). As such, during informed assent/consent, the type of data collected via EARS (e.g., “collect every word that you type on all the apps on your smartphone”), the purpose of collecting these data (e.g., “use smartphones to better understand depression”), and potential risks of participation (e.g., “it’s possible that your information could be lost or stolen, or that your information could not be kept completely private”) have been explained to participants in plain language. The EARS app was also discussed during the initial phone screen before participants were formally consented/assented. Participants were also told that their participation was completely voluntary and that they may withdraw from the study (including just the EARS portion) at any time, even after providing assent/consent.

Second, we have taken several steps to de-identify the key input data. This includes: (a) during EARS installation, no identifying information was entered into the app—only participants’ assigned study ID, (b) the EARS app does not collect anything typed into a secure field (e.g., passwords), and (c) any identifiable information (e.g., date of birth, phone number) was removed using automated algorithms prior to data processing and analysis. Despite our best efforts, there is always a possibility that participants may be re-identified from the key input data. As such, secure data collection and storage are key, which are described below.

Third, the EARS app employs industry-standard encryption protocols and is compliant with the European Union’s General Data Protection Regulation. A detailed description of the data protection procedures can be found in Lind et al., (2018, 2023). Briefly, EARS data are continuously encrypted and uploaded to a secure cloud computing service for storage. After transmission to the cloud, the EARS app then deletes the unencrypted data from participant’s phone. Upon completion of, or withdrawal from, the study, participant’s uninstallation of the EARS app automatically deletes all EARS data still residing on the phone.

Last, only authorized research personnel listed under this study’s IRB can download and unencrypt the key input data, which are then stored in our secure server. To minimize the risk of re-identification, key input data were deleted from our server as soon as data processing was completed. Only unidentifiable, aggregated data were used for analysis and deposited to the online repositories. Note that one of the investigators of this study, Dr. Nicholas Allen, is also the EARS app developer. However, as specified in the IRB, Dr. Allen and his research team cannot use participant’s data for purposes outside the scope of this study (e.g., sell to third parties). We have also noted this conflict of interest in our informed assent/consent form in the spirit of full transparency: “Note that the University of Oregon intends to commercialize EARS technology used in this research. It is possible the University of Oregon and individual investigators involved in this research may financially benefit from the research. However, your data will not be sold under any circumstances.”

**Table S1**

**Demographic and Clinical Characteristics by Inclusion Status**

| Characteristic | | Included  *n* = 126 | Excluded  *n* = 37 | Test statistics | Effect size |
| --- | --- | --- | --- | --- | --- |
| **Demographic** | |  |  |  |  |
| Sex (female) *n* (%) | | 93 (73.8) | 24 (64.9) | χ^2^(1)=0.73 | *V*=0.083 |
| Cisgender *n* (%) | | 117 (92.8) | 37 (100) | Fisher’s exact test *p*=.21 | *V*=0.10 |
| Age *M* (*SD*) | | 16.30 (1.49) | 16.76 (1.50) | *t*(161)=1.63 | *d*=0.30 |
| Race/Ethnicity *n* (%) | |  |  | Fisher’s exact test *p*=.038* | *V*=0.17 |
|  | White | 51 (40.5) | 6 (16.2) |  |  |
|  | Hispanic | 38 (30.2) | 15 (40.5) |  |  |
|  | Asian | 22 (17.5) | 7 (18.9) |  |  |
|  | Black | 9 (7.1) | 6 (16.2) |  |  |
|  | Biracial/Multiracial | 6 (4.8) | 3 (8.1) |  |  |
| Annual household income *n* (%)^a^ | |  |  | Fisher’s exact test *p*=.13 | *V*=0.12 |
|  | < $24,999 | 6 (5.7) | 2 (8.7) |  |  |
|  | $25,000-$49,999 | 13 (12.3) | 4 (17.4) |  |  |
|  | $50,000-$74,999 | 8 (7.5) | 3 (13.0) |  |  |
|  | $75,000-$99,999 | 18 (17.0) | 7 (30.4) |  |  |
|  | ≥ $100,000 | 61 (57.5) | 7 (30.4) |  |  |
| Site (Chicago) *n* (%) | | 71 (56.3) | 7 (18.9) | χ^2^(1)=14.59*** | *V*=0.31 |
| Follow-up length (weeks) *M* (*SD*) | | 48.50 (10.77) | 48.16 (11.03) | *t*(161)=-0.13 | *d*=-0.025 |
| **Clinical** | |  |  |  |  |
| Diagnosis *n* (%) | |  |  | χ^2^(1)=3.03† | *V*=0.15 |
|  | Remitted MDD | 66 (52.4) | 26 (70.3) |  |  |
|  | No lifetime diagnosis | 60 (47.6) | 11 (29.7) |  |  |
| Had an MDE during follow-up *n* (%) | | 29 (23.0) | 13 (35.1) | χ^2^(1)=1.61 | *V*=0.12 |
| Taking psychiatric medication at baseline *n* (%) | | 33 (26.2) | 7 (18.9) | χ^2^(1)=0.47 | *V*=0.071 |
| Baseline CDRS-R *T* score *M* (*SD*) | | 38.45 (8.69) | 39.24 (9.67) | *t*(161)=0.47 | *d*=0.089 |
| Baseline WASI-II Vocabulary *T* score *M* (*SD*) | | 62.13 (8.62) | 59.51 (12.45) | *t*(161)=-1.46 | *d*=-0.27 |

*Note.* MDD = major depressive disorder; MDE = major depressive episode; CDRS-R = Children’s Depression Rating Scale–Revised, WASI-II = Wechsler Abbreviated Scale of Intelligence-II.

^a^ Household income missing/unknown for 27 participants in the included group and 14 participants in the excluded group.

† *p* < .10. * *p* < .05. *** *p* < .001.

**Appendix S2. Supplemental Results**

**Figure S1**

**Survival Curve for Major Depressive Episode During Follow-Up**

| **A. Alpha power**  **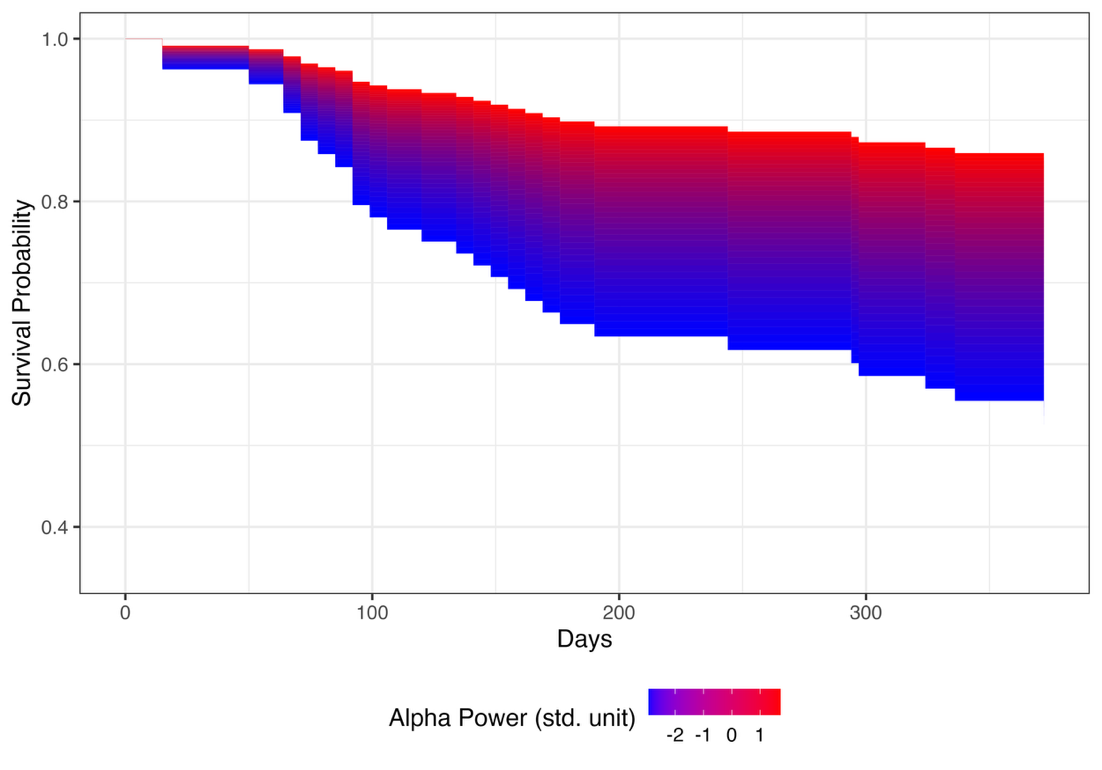** |
| --- |
| **B. First-person singular pronouns**  **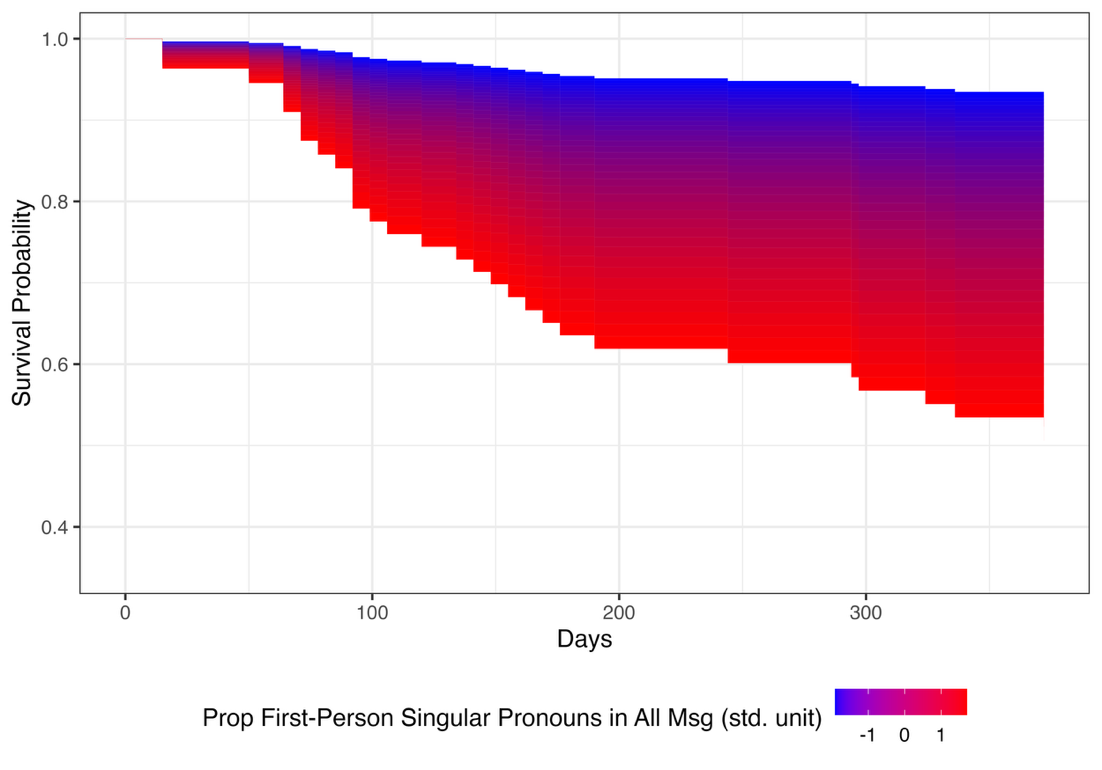** |

*Note.* Figures denote the effect of the predictor on survival probability, estimated in separate models.

**Table S2**

**Prospective Predictors of Risk for Major Depressive Episode**

| Predictor | Estimate | *SE* | *p* | HR | 95% CI | |
| --- | --- | --- | --- | --- | --- | --- |
|  |  |  |  |  | Lower | Upper |
| **Model: Alpha power** |  |  |  |  |  |  |
| Alpha power | -0.24 | 0.075 | .001 | 0.78 | 0.68 | 0.91 |
| Sex (Female) | 1.23 | 0.10 | < .001 | 3.42 | 2.78 | 4.19 |
| Baseline CDRS-R | 0.38 | 0.068 | < .001 | 1.46 | 1.28 | 1.67 |
| **Model: First-person singular pronouns** | | | |  |  |  |
| First-person singular pronouns | 0.70 | 0.19 | < .001 | 2.02 | 1.39 | 2.95 |
| Sex (Female) | 1.17 | 0.24 | < .001 | 3.22 | 2.00 | 5.18 |
| Baseline CDRS-R | 0.46 | 0.018 | < .001 | 1.59 | 1.53 | 1.65 |
| **Model: First-person singular pronouns in negative messages only** | | | | | | |
| First-person singular pronouns | 0.33 | 0.18 | .066 | 1.39 | 0.98 | 1.97 |
| Sex (Female) | 1.16 | 0.11 | < .001 | 3.18 | 2.55 | 3.98 |
| Baseline CDRS-R | 0.38 | 0.038 | < .001 | 1.46 | 1.35 | 1.57 |
| **Model: First-person singular pronouns in positive messages only** | | | | | | |
| First-person singular pronouns | 0.27 | 0.069 | < .001 | 1.31 | 1.14 | 1.50 |
| Sex (Female) | 1.26 | 0.14 | < .001 | 3.52 | 2.69 | 4.61 |
| Baseline CDRS-R | 0.44 | 0.047 | < .001 | 1.56 | 1.42 | 1.70 |
| **Model: Alpha power + first-person singular pronouns** | | | |  |  |  |
| Alpha power | -0.27 | 0.13 | .041 | 0.76 | 0.58 | 0.99 |
| First-person singular pronouns | 0.72 | 0.14 | < .001 | 2.05 | 1.56 | 2.71 |
| Sex (Female) | 1.10 | 0.23 | < .001 | 3.02 | 1.94 | 4.71 |
| Baseline CDRS-R | 0.41 | 0.023 | < .001 | 1.51 | 1.44 | 1.58 |
| **Model: Alpha power + first-person singular pronouns in negative messages only** | | | | | | |
| Alpha power | -0.30 | 0.065 | < .001 | 0.74 | 0.65 | 0.84 |
| First-person singular pronouns | 0.38 | 0.15 | .010 | 1.46 | 1.09 | 1.96 |
| Sex (Female) | 1.13 | 0.10 | < .001 | 3.08 | 2.52 | 3.77 |
| Baseline CDRS-R | 0.34 | 0.050 | < .001 | 1.40 | 1.27 | 1.55 |
| **Model: Alpha power + first-person singular pronouns in positive messages only** | | | | | | |
| Alpha power | -0.25 | 0.098 | .012 | 0.78 | 0.64 | 0.95 |
| First-person singular pronouns | 0.27 | 0.040 | < .001 | 1.31 | 1.21 | 1.42 |
| Sex (Female) | 1.23 | 0.12 | < .001 | 3.41 | 2.70 | 4.30 |
| Baseline CDRS-R | 0.41 | 0.057 | < .001 | 1.51 | 1.35 | 1.69 |

*Note*. HR = hazard ratio. CDRS-R = Children’s Depression Rating Scale–Revised.

**Table S3**

**Baseline Alpha Power Predicting First-Person Singular Pronoun Usage**

| Predictor | β | *SE* | *p* | 95% CI | |
| --- | --- | --- | --- | --- | --- |
|  |  |  |  | Lower | Upper |
| **Model: First-person singular pronouns** | | | |  |  |
| Intercept | -0.20 | 0.069 | .005 | -0.33 | -0.061 |
| Alpha power | 0.17 | 0.059 | .004 | 0.055 | 0.29 |
| Sex (Female) | 0.27 | 0.12 | .024 | 0.036 | 0.50 |
| Baseline CDRS-R | 0.10 | 0.054 | .053 | -0.002 | 0.21 |
| **Model: First-person singular pronouns in negative messages only** | | | | | |
| Intercept | -0.19 | 0.037 | < .001 | -0.27 | -0.12 |
| Alpha power | 0.20 | 0.042 | < .001 | 0.11 | 0.28 |
| Sex (Female) | 0.26 | 0.075 | < .001 | 0.11 | 0.41 |
| Baseline CDRS-R | 0.21 | 0.033 | < .001 | 0.15 | 0.28 |
| **Model: First-person singular pronouns in positive messages only** | | | | | |
| Intercept | 0.054 | 0.017 | .002 | 0.021 | 0.088 |
| Alpha power | 0.16 | 0.056 | .004 | 0.053 | 0.28 |
| Sex (Female) | -0.074 | 0.029 | .013 | -0.13 | -0.016 |
| Baseline CDRS-R | -0.088 | 0.092 | .34 | -0.27 | 0.094 |

*Note*. CDRS-R = Children’s Depression Rating Scale–Revised.

**Examining First-Person Singular Pronoun Usage by Valence of the Messages**

Given that self-focused attention on negatively valenced information may be particularly linked to depression (Collins et al., 2025; Mor & Winquist, 2002; Pyszczynski et al., 1987), we examined the mediation model using first-person singular pronouns calculated only in negative vs. positive messages. Specifically, each message was classified as either positive, negative, or neutral using the TweetNLP package (Camacho-Collados et al., 2022), consisting of a transformer-based language model trained on ~124 million tweets from January 2018 to December 2021. Linguistic self-focused attention was calculated in the same way as described in the main manuscript, except that only positive or negative messages were included in the averaging. It should be noted that, across participants, most messages were classified as neutral (*M* = 70.83%, *SD* = 7.86, range = 48.74-91.89), with an average of 17.44% messages classified as positive (*SD* = 5.90, range = 4.63-35.09) and 11.73% classified as negative (*SD* = 4.51, range = 2.10-23.06). Consequently, the linguistic self-attention scores in valenced messages may be noisy and the results reported below should be considered preliminary.

Mediation models are depicted in Figure S2, with full model outputs shown in Table S2 and S3. Specifically, greater first-person singular pronoun usage in positive messages (HR = 1.31, *p* < .001) and marginally, negative messages (HR = 1.39, *p* = .066) increased risk for MDE during follow-up. Additionally, greater alpha power significantly predicted subsequent first-person singular pronoun usage in both positive (β = 0.16, *p* = .004) and negative messages (β = 0.20, *p* < .001). Mediation models similarly indicated a suppression effect, although not statistically significant, such that accounting for first-person singular pronoun usage in positive or negative messages, the direct effect of alpha power on risk for MDE became stronger than the total effect for which first-person singular pronoun usage was not accounted (positive: HR = 0.78, *p* = .012; negative: HR = 0.74, *p* < .001). Collectively, although results showed similar patterns, the effect sizes were stronger for first-person singular pronoun usage in negative messages than in positive messages.

**Figure S2**

**Mediation Model for the Association Between Resting State Alpha Power and Risk for Major Depressive Episode, Mediated by First-Person Singular Pronoun Usage in Positive vs. Negative Messages**

**A. First-Person Singular Pronouns in Positive Messages Only**


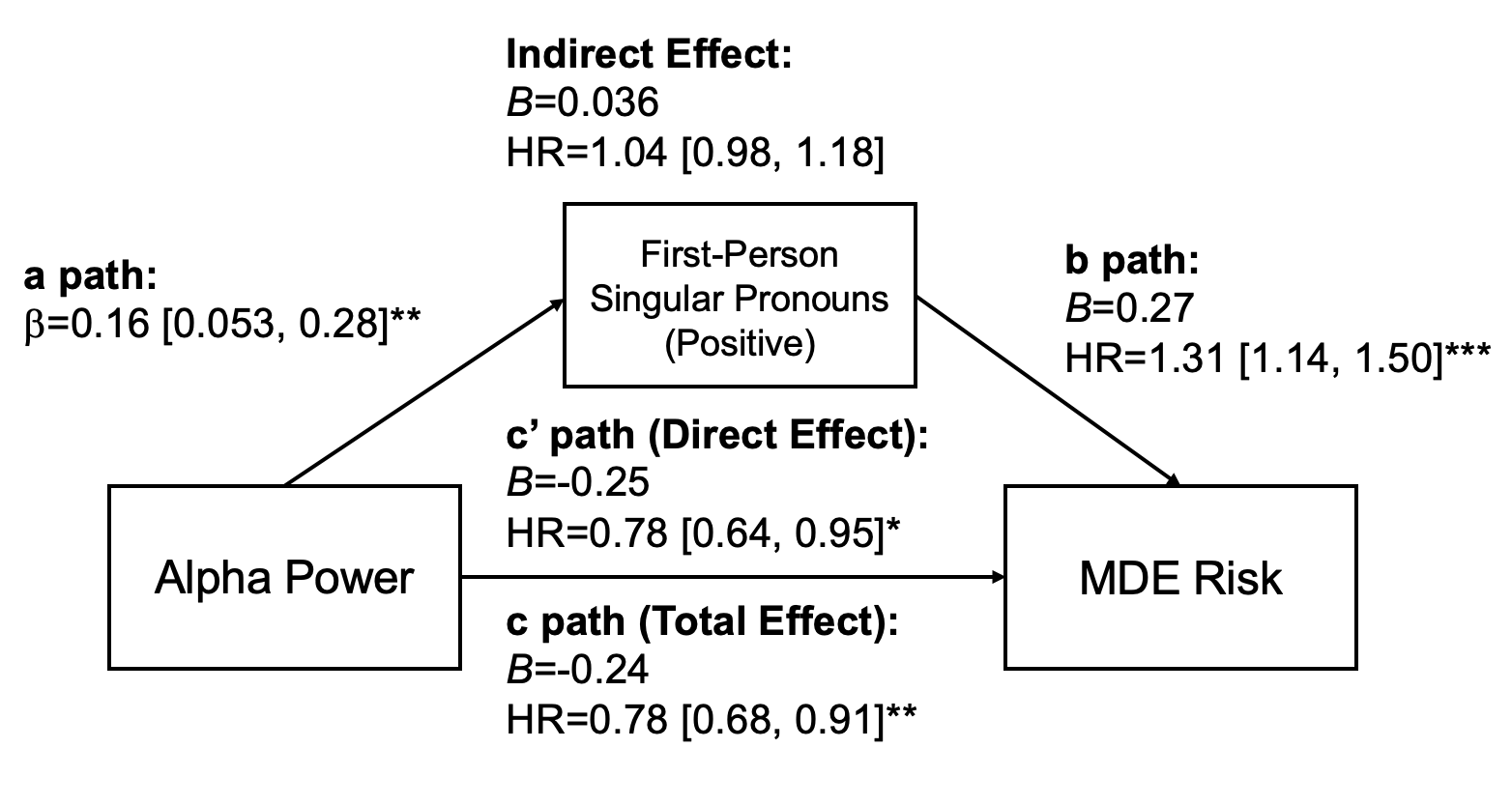


**B. First-Person Singular Pronouns in Negative Messages Only**


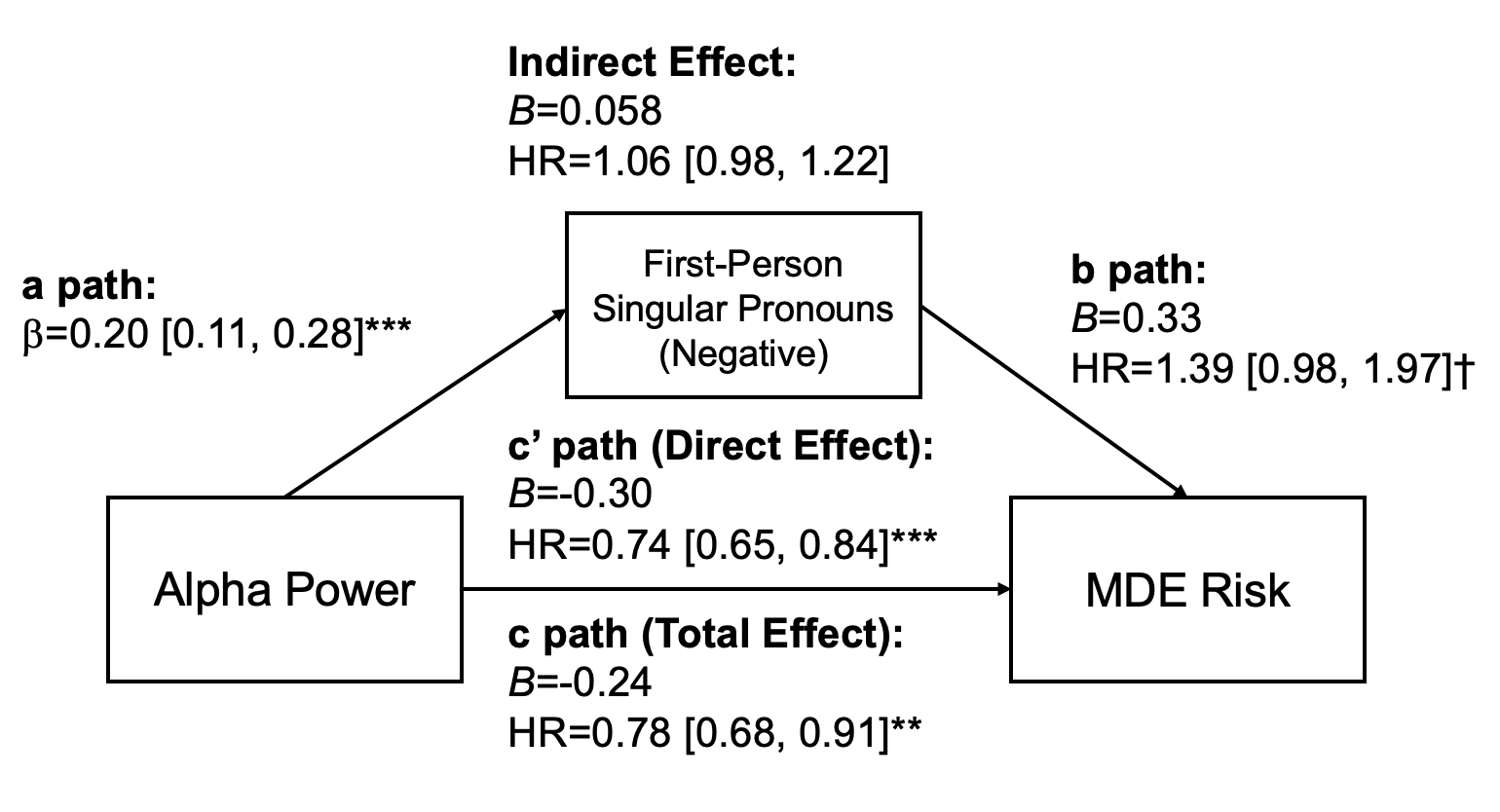


*Note.* All models included the covariates of sex and baseline CDRS-R depression score and cluster robust standard errors. HR = hazard ratio.

† *p* < .10. * *p* < .05. ** *p* < .01. *** *p* < .001.

**Supplemental References**

Bigdely-Shamlo, N., Mullen, T., Kothe, C., Su, K. M., & Robbins, K. A. (2015). The PREP pipeline: Standardized preprocessing for large-scale EEG analysis. *Frontiers in Neuroinformatics*, *9*(JUNE), 1–19. https://doi.org/10.3389/fninf.2015.00016

Camacho-Collados, J., Rezaee, K., Riahi, T., Ushio, A., Loureiro, D., Antypas, D., Boisson, J., Espinosa-Anke, L., Liu, F., Martínez-Cámara, E., Medina, G., Buhrmann, T., Neves, L., & Barbieri, F. (2022). TweetNLP: Cutting-Edge Natural Language Processing for Social Media. In W. Che & E. Shutova (Eds.), *Proceedings of the The 2022 Conference on Empirical Methods in Natural Language Processing: System Demonstrations* (pp. 38–49). Association for Computational Linguistics. https://aclanthology.org/2022.emnlp-demos.5

Collins, A. C., Lekkas, D., Nemesure, M. D., Griffin, T. Z., Price, G. D., Pillai, A., Nepal, S., Heinz, M. V., Campbell, A. T., & Jacobson, N. C. (2025). Semantic signals in self-reference: The detection and prediction of depressive symptoms from the daily diary entries of a sample with major depressive disorder. *Journal of Psychopathology and Clinical Science*. https://doi.org/10.1037/ABN0001003

Delorme, A., & Makeig, S. (2004). EEGLAB: an open source toolbox for analysis of single-trial EEG dynamics including independent component analysis. *Journal of Neuroscience Methods*, *134*, 9–21.

Jacobson, N. C., Bentley, K. H., Walton, A., Wang, S. B., Fortgang, R. G., Millner, A. J., Coombs, G., Rodman, A. M., & Coppersmith, D. D. L. (2020). Ethical dilemmas posed by mobile health and machine learning in psychiatry research. *Bulletin of the World Health Organization*, *98*(4), 270–276. https://doi.org/10.2471/BLT.19.237107

Kothe, C. A., & Makeig, S. (2013). BCILAB: A platform for brain-computer interface development. *Journal of Neural Engineering*, *10*(5), 56014–56031. https://doi.org/10.1088/1741-2560/10/5/056014

Lind, M. N., Byrne, M. L., Wicks, G., Smidt, A. M., & Allen, N. B. (2018). The Effortless Assessment of Risk States (EARS) Tool: An Interpersonal Approach to Mobile Sensing. *JMIR Mental Health*, *5*(3), e10334. https://doi.org/10.2196/10334

Lind, M. N., Kahn, L. E., Crowley, R., Reed, W., Wicks, G., & Allen, N. B. (2023). Re-introducing the Effortless Assessment Research System (EARS). *JMIR Mental Health*, *10*(1), e38920. https://doi.org/10.2196/38920

Mohr, D. C., Shilton, K., & Hotopf, M. (2020). Digital phenotyping, behavioral sensing, or personal sensing: names and transparency in the digital age. *Npj Digital Medicine*, *3*(1), 1–2. https://doi.org/10.1038/s41746-020-0251-5

Mor, N., & Winquist, J. (2002). Self-focused attention and negative affect: A meta-analysis. *Psychological Bulletin*, *128*(4), 638–662. https://doi.org/10.1037/0033-2909.128.4.638

Mullen, T. R., Kothe, C. A. E., Chi, Y. M., Ojeda, A., Kerth, T., Makeig, S., Jung, T. P., & Cauwenberghs, G. (2015). Real-time neuroimaging and cognitive monitoring using wearable dry EEG. *IEEE Transactions on Biomedical Engineering*, *62*(11), 2553–2567. https://doi.org/10.1109/TBME.2015.2481482

Pion-Tonachini, L., Kreutz-Delgado, K., & Makeig, S. (2019). ICLabel: An automated electroencephalographic independent component classifier, dataset, and website. *NeuroImage*, *198*, 181–197. https://doi.org/10.1016/j.neuroimage.2019.05.026

Pyszczynski, T., Arkowitz, H., Beckman, J., Brehm, J., Carver, C., Gollwitzer, P., Hamilton, J., & Weisz, J. (1987). Self-Regulatory Perseveration and the Depressive Self-Focusing Style: A Self-Awareness Theory of Reactive Depression. *Psychological Bulletin*, *102*(1), 122–138.

Vitak, J., Shilton, K., & Ashktorab, Z. (2016). Beyond the Belmont principles: Ethical challenges, practices, and beliefs in the online data research community. *Proceedings of the ACM Conference on Computer Supported Cooperative Work, CSCW*, *27*, 941–953. https://doi.org/10.1145/2818048.2820078
